# Biocatalytic hydrogenation of the C=C bond in the enone unit of hydroxylated chalcones—process arising from cyanobacterial adaptations

**DOI:** 10.1007/s00253-018-9109-z

**Published:** 2018-06-04

**Authors:** Beata Żyszka-Haberecht, Anna Poliwoda, Jacek Lipok

**Affiliations:** Department of Analytical and Ecological Chemistry, Faculty of Chemistry, University of Opole, Oleska 48, 45-052 Opole, Poland

**Keywords:** Hydroxylated chalcones, Biocatalysis, Cyanobacteria, Regiospecific hydrogenation

## Abstract

To verify the hypothesis that cyanobacteria naturally biosynthesising polyphenolic compounds possess an active enzymatic system that enables them to transform these substances, such an ability of the biocatalytic systems of whole cells of these biota was assessed for the first time. One halophilic strain and seven freshwater strains of cyanobacteria representing four of the five taxonomic orders of Cyanophyta were examined to determine the following: (i) whether they contain polyphenols, including flavonoids; (ii) the resistance of their cultures when suppressed by the presence of exogenous hydroxychalcones—precursors of flavonoid biosynthesis and (iii) whether these photoautotrophs can transform hydroxylated chalcones. All examined strains were found to contain polyphenols and flavonoids, and the growth of their cultures was inhibited in the presence of 2′-hydroxychalcone, 2″-hydroxychalcone and 4″-hydroxychalcone. We also confirmed that the examined cyanobacteria transformed hydroxychalcones via hydrogenative bio-reduction and formed the corresponding hydroxydihydro derivatives with yields above 90% whenever the substrates were bioavailable for such a conversion. Moreover, we observed that the routes and efficiency of biohydrogenation (and hydroxylation) of chalcones were dependent on the location of the hydroxyl substituent. The final products obtained as the results of biotransformations were extracted from the media and identified by mass spectrometry (LC-MS/MS) and nuclear magnetic resonance (^1^H NMR, ^13^C NMR, COSY, HSQC). Based on those results, we believe that the very efficient biohydrogenation of hydroxychalcones, which may easy be scaled up for biotechnological purposes, reflects the natural activity of the cyanobacterial defence system, because hydroxydihydrochalcones were less active inhibitors of the growth of cyanobacterial cultures than the corresponding substrates.

## Introduction

Chalcones are a group of plant-derived compounds belonging to the flavonoid family that are synthesised through the phenylpropanoid pathway and play a vital role in different metabolic pathways in photosynthesising organisms. Structurally, chalcones are composed of two aryl groups (A- and B-rings) linked by an open-chain three-carbon unit α,β-unsaturated carbonyl system. Naturally occurring chalcones are broadly distributed in various plant species and possess a conspicuously yellow colour (Attar et al. 2011; Ballester et al. 2010). They are biogenetic precursors of all known flavonoids and exist most commonly in hydroxylated form. Chalcones are frequently produced in nature by hydroxylases in the biosynthetic pathways of plants (Ni et al. 2004). In view of their relative structural simplicity and associated facility of synthesis, coupled with their attractive biological activities (Avila et al. 2008; Karaman et al. 2010), chalcones continue to enjoy considerable attention from chemists researching new molecular scaffolds for the creation of novel therapeutics (Okoniewska et al. 2016; Ritter et al. 2014; Stompor et al. 2015).

Biocatalysis represents a successful and, in many cases, outstanding substitute to standard chemical synthesis (Park et al. 2009) or extraction of chalcones from plant material (Du et al. 2010). This strategy is particularly valuable for microbial whole cell systems, which afford cheaper, scalable, and more available means of efficiently acquiring derivatives of chalcones (de Carvalho and da Fonseca 2006). Thus far, transformations of hydroxylated chalcones by whole cell systems have been performed only by heterotrophic microorganisms and the main reactions of these processes mimic the natural metabolic conversion pathways of these molecules in plant kingdom. Therefore, hydrogenation, dehydrogenation, hydroxylation, O-methylation, O-demethylation, glycosylation, deglycosylation, C ring cleavage of the benzo-γ-pyrone system, cyclization and carbonyl reduction were found as the main ways of transformation (Cao et al. 2015). Microbial conversion of compounds containing a three-carbon enone moiety has attracted increasing attention, particularly with the recent rediscovery of ene-reductases from the old yellow enzyme family. These flavoenzymes selectively catalyse the hydrogenation of activated C=C bonds of α,β-unsaturated carbonyl molecules in excellent yield (Fu et al. 2013). Ene-reductases have also been identified in prokaryotic microalgae, i.e. cyanobacteria (Fu et al. 2013), and used for light-induced, photocatalytic reduction of C=C bonds (Koninger et al. 2016) that conduces to the formation of dihydrochalcones. Because of their high sweet taste, these compounds have potential applications in the food industry for the production of low-calorie, multicomponent, nontoxic and safety sweeteners of natural origin. Consequently, their UV-protective, antioxidant and health-promoting properties make dihydrochalcones of interest to the pharmaceutical and cosmetics industries (DuBois et al. 1977; Winnig et al. 2007).

Biocatalytic transformations using microorganisms are anticipated to be more selective and environmentally friendly (Matsuda et al. 2009; Nakamura et al. 2003); however, the microbes do not always deliver the expected reactions. The major disadvantage of bioconversion is the prerequisite for an aqueous ambience for most enzymatic reactions, which can be a limitation for the slightly soluble organic compounds acted as substrates. Additionally, a real difficulty associated with the use of biocatalysts is the successive supply of the cofactors NADH or NADPH, which deliver reducing power and chemical energy. In case of oxygenic photoautotrophic organisms, the solar light energy captured by photosynthetic systems is converted to electrochemical energy to regenerate NADPH from NADP^+^ via photosynthetic electron-transfer reactions. NADPH is the solitary reducing agent for the reduction of exogenous artificial substrates (Nakamura and Yamanaka 2002). Therefore, living cyanobacterial cells that possess the ability to regenerate their own respective cofactors are hopeful and eminently effective pretender for novel ‘photobiocatalysts’. Additionally, cyanobacterial species possess thylakoids, an intracellular membrane system that makes them highly suitable hosts for expressing the P450 enzymes that participate in phenylpropanoids biosynthetic (Melis 1999; Xue and He 2015). As prokaryotic microalgae, cyanobacteria are an unconventional and attractive source of phenylpropanoids and create great opportunities for discovering unique secondary metabolites with potential biological activities due to the facility of mass-scale cultivation. Moreover, several inherent features of cyanobacteria, such as oxygenic photosynthesis, amenability to genetic engineering and capacity to survive in extreme habitats or under chemical stress, make them prominent cell factories for targeted biotransformations (Xue and He 2015). We hypothesise then that organisms that produce phenylpropanoids, which are also precursors of flavonoids (Goiris et al. 2014; Singh et al. 2017a), must possess active enzymatic pathways that enable them to transform these compounds according to cellular needs.

To verify this hypothesis, the ability of the biocatalytic systems of whole cells of halophilic and freshwater species to transform hydroxylated chalcones was studied. All experiments were performed in batch cultures on both analytical and preparative scales under conditions mimicking their environmental response.

## Materials and methods

### Chemicals

The substrate 2′-hydroxychalcone was obtained following the method of Yadav et al. (Yadav et al. 2012). The substrate 2″-hydroxychalcone (cat. no. 513067) was purchased from Sigma-Aldrich (Poznan, Poland). The synthesis of 4″-hydroxychalcone was carried out according to the literature method (Tran et al. 2009) under strongly basic conditions (KOH) using methanol as a solvent. All hydroxylated chalcone stock solutions were prepared in dimethyl sulfoxide (DMSO) (15 mg/mL) and sterilised by filtration prior to their addition to the medium. All stock solutions were made on the same day of use and kept in dark to prevent photodamage (Zyszka et al. 2017a). All components of the medium were purchased from Avantor Performance Materials Poland S.A. (Gliwice, Poland). Other chemicals were of analytical grade purity, purchased from commercial suppliers: Sigma-Aldrich, Fluka and Avantor Performance Materials Poland S.A. All the solutions for culture preparation and maintenance used in this study were prepared using water from Milli-Q water (Merck, Millipore, Germany), whereas special Millipore Milli-Q A10 water of high purity was used for analytical purposes.

### Cyanobacterial strains and culture conditions

One halophilic strain of cyanobacteria, *Spirulina platensis* [strain C1 (PCC9438)], and seven freshwater strains, including *Anabaena sp*. [strain CCALA 007], *Anabaena laxa* [strain CCALA 805], *Aphanizomenon klebahnii* [CCALA 009], *Nodularia moravica* [strain CCALA 797], *Chroococcus minutus* [strain CCALA 055], *Merismopedia glauca* [strain CCALA 099] and *Synechocystis aquatilis* [strain CCALA 190], were used in our experiments. The axenic strain of *Spirulina platensis* was purchased from the Pasteur Culture Collection (PCC) (Institute Pasteur, Paris), whereas all freshwater strains were obtained from the Culture Collection of Autotrophic Organisms (CCALA) (Institute of Botany of the Academy of Sciences, Czech Republic).

Subcultures of cyanobacteria were revitalised every 3 weeks by transferring 10-mL aliquots to 50 mL of fresh suitable media (Forlani et al. 2011). To prepare the inoculates used to initiate the experimental cultures, all tested cyanobacteria were pre-grown in standard media: MSp (ATCC 1679) medium (pH 9.5) for the halophilic strain and BG11 (ATCC 616) or Z8 medium (pH 7.0) for the freshwater strains. The cultures of the tested cyanobacteria were grown at 24 ± 1 °C with 16-h day (1000 lx light intensity) and 8-h night photoperiods, corresponding to the conditions of a long day, in 250-mL Erlenmeyer flasks containing 60 mL of each culture (Allen 1968; Rippka et al. 1979; Żyszka et al. 2017a).

### Searching for natural phenolics in cyanobacterial cells

Biomass samples of all tested cyanobacterial strains were harvested at the end of logarithmic phase (21-day-old subcultures) by centrifugation at 5000×*g* for 1 min. The pellet was washed twice with distilled water to remove adhering components of the medium, and subsequently, the cyanobacterial cells were immediately freeze-dried at − 50 °C and stored at − 20 °C prior to extraction.

For the efficient extraction of polyphenols and flavonoids, 100 mg of lyophilized cells of each strain was sonicated for 30 min in 3 mL of pure methanol using an ultrasonic bath (40 kHz, 100 W; Branson, Danbury, Ct, USA) and for a further 30 min using a Hielscher UP200HT ultrasonic homogeniser (26 kHz, 200 W, Hielscher Ultrasonics GmbH, Teltow, Germany). The homogenates were centrifuged at 5000×*g* for 1 min, and the supernatants were filtered using Whatman No. 1 filter paper, pooled, concentrated to 1 mL and sterilised by filtration using a 0.22-μm Millipore filter. The resultant extracts were stored at 4 °C in the dark for 24 h before determination of total phenolic and flavonoid content.

### Total phenolic assay

The total phenolic content (TPC) was determined according to a modified Folin-Ciocalteu method (Singleton et al. 1999). An aliquot (100 μL) of extracts or standard solution of gallic acid (50, 100, 200, 400, 600 and 1000 μg/ml) was added to a 10-mL volumetric flask and mixed with 0.5 mL of Folin-Ciocalteu phenol reagent. Then, 1 mL of H_2_O was added to the mixture and shaken vigorously. After a 2-min incubation at room temperature, 1.5 mL of Na_2_CO_3_ (20%, *w*/*v*) solution was added, and the volume was then made up to the mark with distilled water. The samples were incubated for 2 h in the dark. The mixture was allowed to stand for 2 h with intermittent shaking. After incubation for 120 min at room temperature in the darkness, the absorbance against distilled water was determined at 760 nm with a UV-Visible spectrophotometer. TPC was expressed as milligram gallic acid equivalents/g dry-extract weight (mg GAE/g DW). A reagent blank was prepared using distilled water.

### Total flavonoid assay

The total flavonoid content (TFC) was determined by the aluminium chloride colorimetric assay (Zhishen et al. 1999). An aliquot (1 mL) of extracts or standard solutions of quercetin (6.25, 12.5, 25, 50, 80 and 100 μg/mL) was added to a 10-mL volumetric flask containing 4 mL of distilled water. To the flask was added 0.30 ml of 5% NaNO_2_, and after 5 min, 0.3 mL of 10% AlCl_3_ was added. After 5 min, 2 mL of 1 M NaOH was added, and the volume was made up to 10 mL with distilled water. The solution was mixed and incubated for 15 min in the dark, and the absorbance was then measured against the blank at 510 nm. The results were expressed as mg quercetin equivalents/g dry-extract weight (mg QE/g DW).

### Determination of the nature of phenolic compounds present in cyanobacterial cells

In order to confirm the presence of phenolic compounds in the cyanobacterial cells and determine the nature of these substances, liquid chromatography coupled with spectrophotometric and mass spectrometry detection (UHPLC-UV-MS/MS) was used for chemical screening of extracts of tested cyanobacterial cells. For this purpose, 500 mg of lyophilized cells of each strain was homogenised in a ceramic mortar and sonicated for 15 min in pure methanol (5 mL) using an Ultrasonic sonicator (40 kHz, 100 W; Branson, Danbury, CT, USA). Then, the samples were centrifuged for 3 min at 3000×*g*, and the supernatant was separated and collected for combination with the next fraction extracted in the same way from the cell debris with 5 mL of fresh methanol. Both methanol extracts were pooled and concentrated to 5 mL under a stream of nitrogen. To avoid contamination of the mass spectrometer and to reduce matrix effects, chlorophylls and carotenoids were largely removed from the extracts by adsorbing these components on an OASIS® MCX ion exchange column (6 mL, 200 of mg sorbent, Waters, Milford, MA, USA). After conditioning the column with 5 mL of methanol, the concentrated cell-free extract was loaded, and the analytes of interest were selectively eluted using 5 mL of methanol (Goiris et al. 2014).

The cell-free extracts were dried under a stream of nitrogen, redissolved in a mixture of 600 μL of methanol and 400 μL of formate buffer (4 g of ammonium formate and 300 μL of formic acid per litre of buffer) and centrifuged (10 min, 13,000×*g*). The samples were then subjected to UHPLC-UV-ESI-MS analysis employing a Dionex UltiMate 3000 UHPLC system (Sunnyvale, CA, USA) coupled to a micrOTOF-QII mass spectrometer (Bruker Daltonics, Germany). Chromatographic separation was carried out on a Gemini-NX C18 column (150 mm × 4.6 mm i.d. 3μm, 110 Å) thermostated at 30° and protected by a Gemini NX pre-column (4 × 3 mm). The mobile phase consisted of acetonitrile with 0.1% formic acid *v/v* (eluent A) and water containing 0.1% formic acid *v/v* (eluent B) delivered by a gradient at a flow rate 250 μL min^−1^. The program for gradient elution was 40% A/60% B (0 min), 100% A/0%B (15 min), 100% A/0% B (20 min), 40% A/60% B (25 min) and 40% A/60% B (30 min). The injection volume was 20 μl. The autosampler temperature was set at 4 °C, and the presence of polyphenols was monitored at 254 nm.

ESI mass spectra were recorded in positive and negative full scan mode. The electrospray ionisation source parameters were as follows: capillary voltage, 3.5 kV; nebuliser pressure, 1.2 bar; drying temperature, 200 °C; dry gas flow rate, 8.0 L min^−1^. Nitrogen was used as both the nebulising and collision gas. Spectra were recorded in positive and negative modes. Full-scan spectra were acquired in the range of *m/z* 50–1000.

Polyphenols were characterised and identified according to accurate measurements of the mass parent ions and fragments during MS/MS analysis using Metabolite Detect 2.0 software (Bruker Daltonics, Germany).

### Determination of the growth of microorganisms in the presence of hydroxylated chalcones

In order to determine the highest concentration of hydroxylated chalcones that did not induce the sudden death of cyanobacterial cells (Żyszka et al. 2017b), a set of screening experiments performed in a concentration range from 5 up to 100 mg/L was arranged. Based on the results of these experiments, a concentration of 20 mg/L was chosen.

The growth of the examined photoautotrophs was assessed by time-course measurements of total chlorophyll content in experimental cultures, as described in our previous paper (Żyszka et al. 2017b). Briefly, representative samples of the cells were obtained by centrifugation, then re-suspended in methanol in order to extract chlorophyll. The content of this photosynthetic dye was determined spectrophotometrically, with the use of Arnon’s formula. Basing on the average levels of chlorophyll in each experimental or control culture replicate, the growth curves were generated and the growth rates of the examined photoautotrophs were calculated. Finally, the ratio of the growth rates of the experimental cultures with respect to the appropriate controls were determined to illustrate the differences in the dynamics of cyanobacterial growth dependent on the strain and the examined hydroxylated chalcone. The percentage values of the ratios of the growth rates of the appropriate strain were correlated with the appropriate compound and presented as the heat map in the results.

### Analytical scale biotransformations of hydroxylated chalcones

Screening (analytical) scale biotransformations of all hydroxylated chalcones were studied according to previously described methodology (Żyszka et al. 2017a). The cyanobacterial inoculum was added to 100-mL Erlenmeyer flasks containing 30 mL of the respective culture medium supplemented with the appropriate hydroxylated chalcone stock solution (0.13%, *v*/*v*) to obtain a final concentration of 20 mg/L of hydroxylated chalcone. The stability of the tested chalcones was positively verified in appropriate substrate control samples consisting of the solution of the tested flavonoid in sterile cultivation medium, whereas the culture controls were established as consisted of cyanobacterial cells in medium cultivated without the hydroxylated chalcone. All experiments, including the controls, were performed at least in triplicate and were incubated under adjusted conditions of light and temperature for 14 days. After this time, the cells and culture media were separated by filtration followed by centrifugation, and each repetition was extracted three times with 10 mL of ethyl acetate. Then, these extracts were combined, dried over anhydrous magnesium sulphate, and the solvent was removed using a vacuum evaporator. The remaining residue dissolved in 200 μL of methanol was subjected to analysis by high-performance planar chromatography (HPTLC) and liquid chromatography-mass spectrometry (LC-MS). The resulting biotransformation yield was established as the average of the values calculated based on the quantification of the products obtained with respect to the areas of relevant peaks.

### Determination of the biotransformation course

The products of biotransformation were identified by HPTLC and UHPLC-UV-ESI-MS techniques similarly as it was reported for dihydrochalcone (Żyszka et al. 2017a). To examine the course of biotransformation, the products of this process were separated in HPTLC experiments, on TLC aluminium plates using a CAMAG Linomat 5 applicator (CAMAG, Muttenz, Switzerland). To verify the presence of expected derivatives of flavonoids, the plates were sprayed with appropriate cerium phosphomolybdate reagent and were gently heated until coloured spots appeared. The retention coefficients (R_f_) and colours of the spots were recorded using CAMAG VisionCats software.

The changes in the chemical composition of the biotransformation media towards the transformation of the tested chalcones were studied by UHPLC-UV-ESI-MS using the same analytical strategy as for the determination of the chemical nature of the phenolics contained in the cells of the studied cyanobacteria.

### Preparative-scale biotransformations of hydroxylated chalcones

Eight repetitions of the same experiment were performed in 1000-mL Erlenmeyer flasks containing 250 mL of the cultures to scale-up the biotransformation process. After 14 days of incubation, the mixtures were extracted with ethyl acetate (each repetition, 3 × 80 mL), dried over anhydrous MgSO_4_ and the solvent was removed. The products of transformation were separated by preparative thin layer chromatography (PTLC) using a CAMAG system and silica gel glass plates without a fluorescent indicator and were extracted with ethyl acetate, which was then evaporated under a stream of nitrogen. Then, the structures of the isolated products were determined and confirmed by mass spectrometry (MS) and nuclear magnetic resonance techniques: ^1^H NMR and ^13^C NMR, including COSY and HSQC correlation experiments. The NMR spectra were measured in DMSO-d6 using a Bruker UltraShield 400 MHz spectrometer with tetramethylsilane (TMS) as an internal reference.

## Results

### Phenolic composition of tested cyanobacterial species

**Fig. 1:**
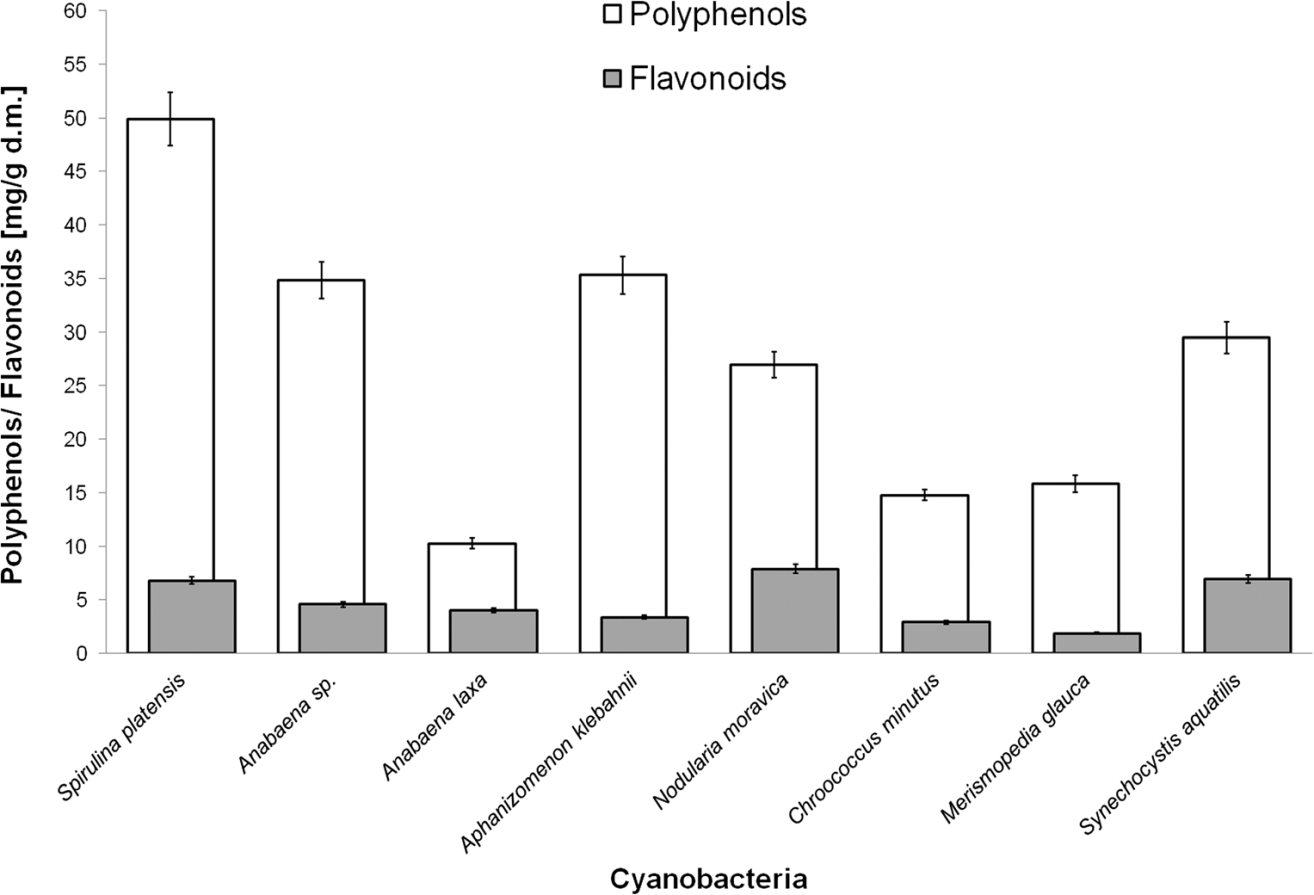
Total phenol and flavonoid content of the tested cyanobacterial cell-free extracts

We tested the hypothesis that cyanobacterial cells naturally contain phenolic compounds and therefore possess the enzymatic apparatus enabling them to transform these compounds. Cell-free extracts of eight strains of cyanobacteria belonging to different genera, including one halophilic strain (*S*. *platensis*) and seven freshwater strains (*A*. *laxa*, *Anabaena* sp., *A*. *klebahnii*, *N*. *moravica*, *C*. *minutus*, *M*. *glauca* and *S*. *aquatilis*), were evaluated for the total content of phenolics (TPC) and flavonoids (TFC). The total amounts of phenolic compounds, including flavonoids, were determined spectrophotometrically, and the results showed that the phenolic composition differed significantly among the strains (Fig. 1). The TPC varied from 10.23 ± 0.51 to 49.87 ± 2.49 mg/g GAE, and the total flavonoid content ranged between 1.87 ± 0.09 and 7.90 ± 0.40 mg/g QE of the extracts. The cyanobacterium *S*. *platensis*, which is well-known for its nutraceutical features, was the richest in phenolic compounds among the examined strains. Relatively high values of TPC were also observed in *A*. *klebahnii*, *Anabaena* sp., *S*. *aquatilis* and *N*. *moravica*, whereas the lowest concentration was observed in the extract of *A*. *laxa*. With respect to flavonoid content, *S*. *platensis* was ranked only third (6.80 ± 0.34 mg/g QE), with higher contents in *N*. *moravica* and *S*. *aquatilis*, which contained 7.90 ± 0.40 and 6.94 ± 0.35 mg/g QE, respectively. The lowest TFC among the tested strains was found in cells of *M*. *glauca*. The UHPLC-MS/MS analysis allowed us to determine the chemical nature of the phenolic compounds extracted from the cyanobacterial biomass. Using the set of standards to fortify appropriate samples, we confirmed the natural presence of phenolic acids, including protocatechuic, gallic, ferulic and sinapic acid, as well as the presence of flavonoids, including apigenin, genistein, naringenin, acacetin, biochanin A, quercetin and dihydroquercetin, in all the studied microalgal strains. Therefore, we surmised that the examined photoautotrophic microorganisms also possess a pool of adequate enzymes required for the biosynthesis and transformation of these compounds with prominent antioxidant properties.

### The sensitivity of cyanobacterial species to hydroxylated chalcones

To verify the assumption about the influence of exogenous hydroxylated chalcones, the growth of these microorganisms was examined in terms of chlorophyll content, which is a determinant of the metabolic activity of phototrophs, at the time of harvest in exponential phase.

**Fig. 2:**
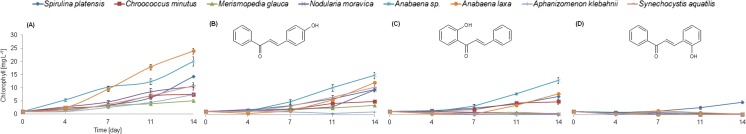
Influence of hydroxylated chalcones, **b** 4″-hydroxychalcone, **c** 2′-hydroxychalcone, **d** 2″-hydroxychalcone, on the growth of cyanobacterial species. **a** Appropriate control cultures without hydroxylated chalcones

The influences of structurally similar hydroxylated chalcones differing only in the location of one hydroxyl group at the aromatic ring, i.e. 2′-hydroxychalcone, 2″-hydroxychalcone, and 4″-hydroxychalcone, on the growth of the eight studied cyanobacteria are illustrated in Fig. 2.

**Fig. 3:**
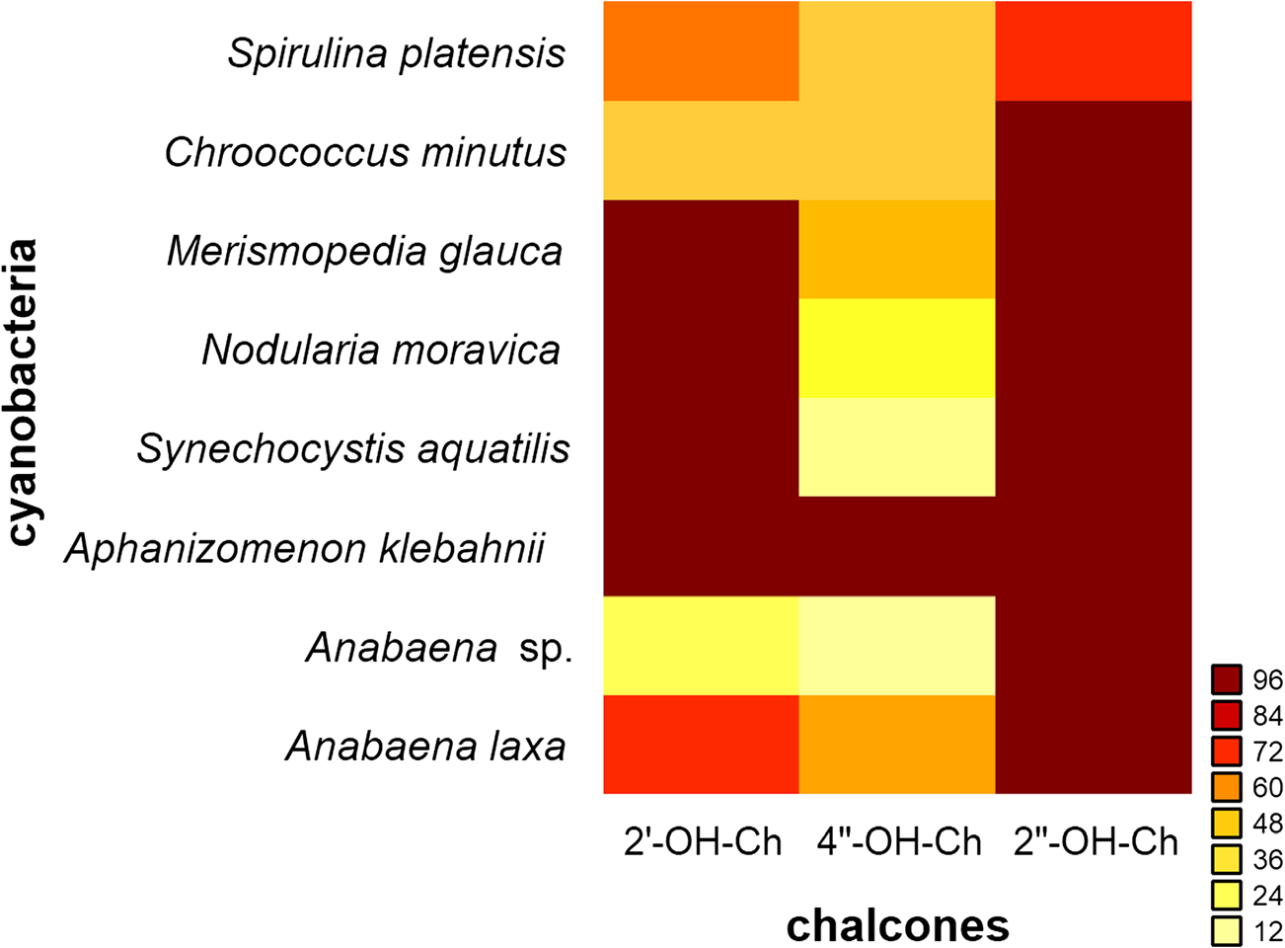
Heat map depicting the intensity of cyanobacterial growth inhibition under the influence of hydroxylated chalcones. High-intensity (red) cells indicate a high inhibitory effect, and low-intensity (yellow) cells indicate a low inhibitory effect

There was marked variation in the content of total chlorophyll in the cyanobacterial strains (Fig. 2a). The maximum content of chlorophyll was observed in *A*. *laxa*, whereas the minimum content of chlorophyll was recorded in *M*. *glauca*. The halophilic strain, *S*. *platensis*, was generally more resistant to the tested hydroxylated chalcones than the freshwater cyanobacteria, although the level of sensitivity observed was dependent on the chemical. 2″-Hydroxychalcone inhibited the growth of the cyanobacterial species most effectively (Fig. 2d). The growth of all freshwater species stopped completely after 4 days of culture, whereas the growth rate of *S*. *platensis* was reduced by 72% during the 14 days of the experiment compared with the appropriate control culture. By contrast, 2′-hydroxychalcone (in fact, the mixture of 2′-hydroxychalcone and flavanone) affected cyanobacterial growth in a species-specific manner (Fig. 2c). This compound entirely inhibited the growth of the freshwater cyanobacteria *M*. *glauca*, *N*. *moravica*, *S*. *aquatilis* and *A*. *klebahnii* but only limited the growth rate of *S*. *platensis* and *A*. *laxa* by 62 and 69%, respectively. The most resistant cyanobacteria against 2′-hydroxychalcone were *Ch*. *minutus* and *Anabaena* sp., whose growth was suppressed by 42 and 22%, respectively. 4″-Hydroxychalcone had the lowest negative influence on the examined strains, as the cultures of *S*. *aquatilis* and *Anabaena* sp. were suppressed by only 15 and 11%, respectively. Although the growth of *A*. *klebahnii* was completely inhibited, the growth of other strains was reduced by approximately 40 to 53% (Fig. 2b). The effects of the hydroxylated chalcones on the cyanobacterial strains was apparent in less than 4 days from the moment of supplementation, indicating efficient transport of these compounds into the cells (Żyszka et al. 2017b). The heat map presented in Fig. 3 summarises the growth inhibition patterns of these compounds as characterised by the ratios of the growth rates of the experimental cultures to the growth rates of the appropriate controls, expressed as percentage values.

### Biocatalytic transformations of hydroxylated chalcones by cyanobacteria

To understand the interactions between the hydroxylated chalcones and cyanobacteria more deeply, the fate of the tested compounds, 2′-hydroxychalcone (1), 2″-hydroxychalcone (2) and 4″-hydroxychalcone (3), which all contain the three-carbon enone moiety 1,3-diphenyl-2-propen-1-one, in biocatalytic transformations conducted by the suppressed cyanobacteria was studied.

### (Bio)conversions of 2′-hydroxychalcone (1)

**Fig. 4:**
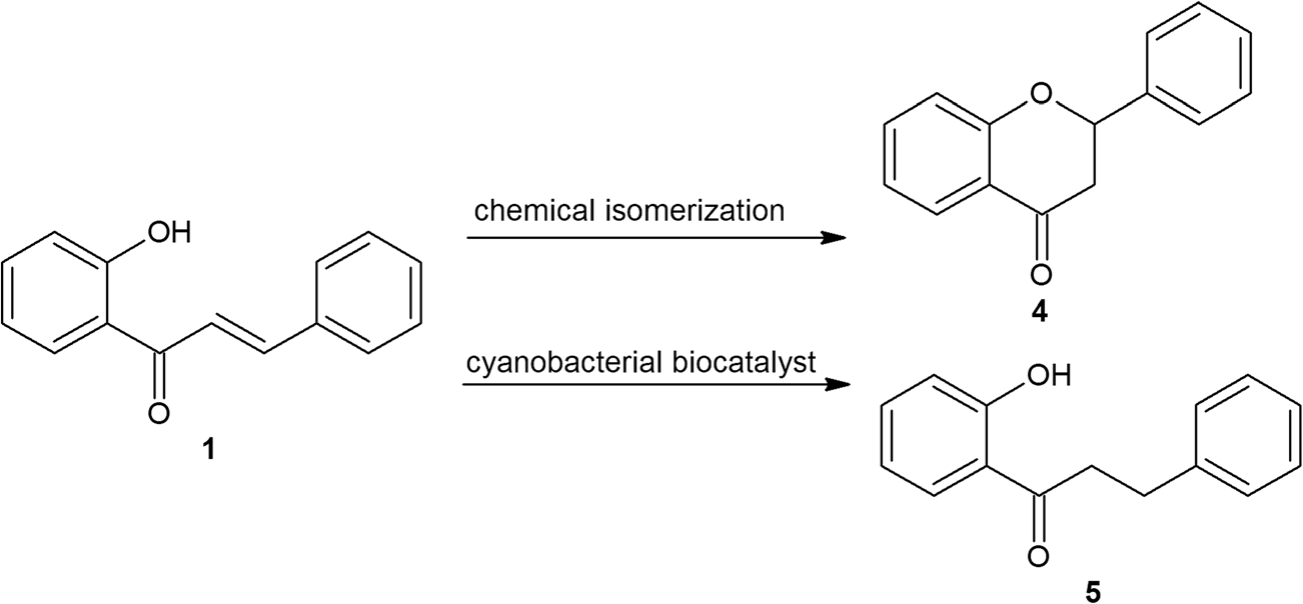
Conversion of 2′-hydroxychalcone to flavanone (4) and 2′-hydroxydihydrochalcone (5)

Two products of transformation of 2′-hydroxychalcone were obtained in quantifiable amounts. According to concentration, the first was flavanone (4), whereas 2′-hydroxydihydrochalcone (5) was second (Fig. 4).

The nature of the observed conversions was very interesting. 2′-Hydroxychalcone was not fully accessible to the cyanobacterial catalysts during cultivation because this compound had already undergone isomerisation to flavanone via intramolecular rearrangement at the time of introduction to each of the used culture media. This process occurs under fully environmentally friendly conditions (microbial medium at room temperature) and strongly favours the formation of flavanone, since this substance was detected at a ratio of 20:1 over the initial 2′-hydroxychalcone. This conversion seems to be natural for 2′-hydroxychalcone because the presence of flavanone was even confirmed in the purchased standard.

**Fig. 5:**
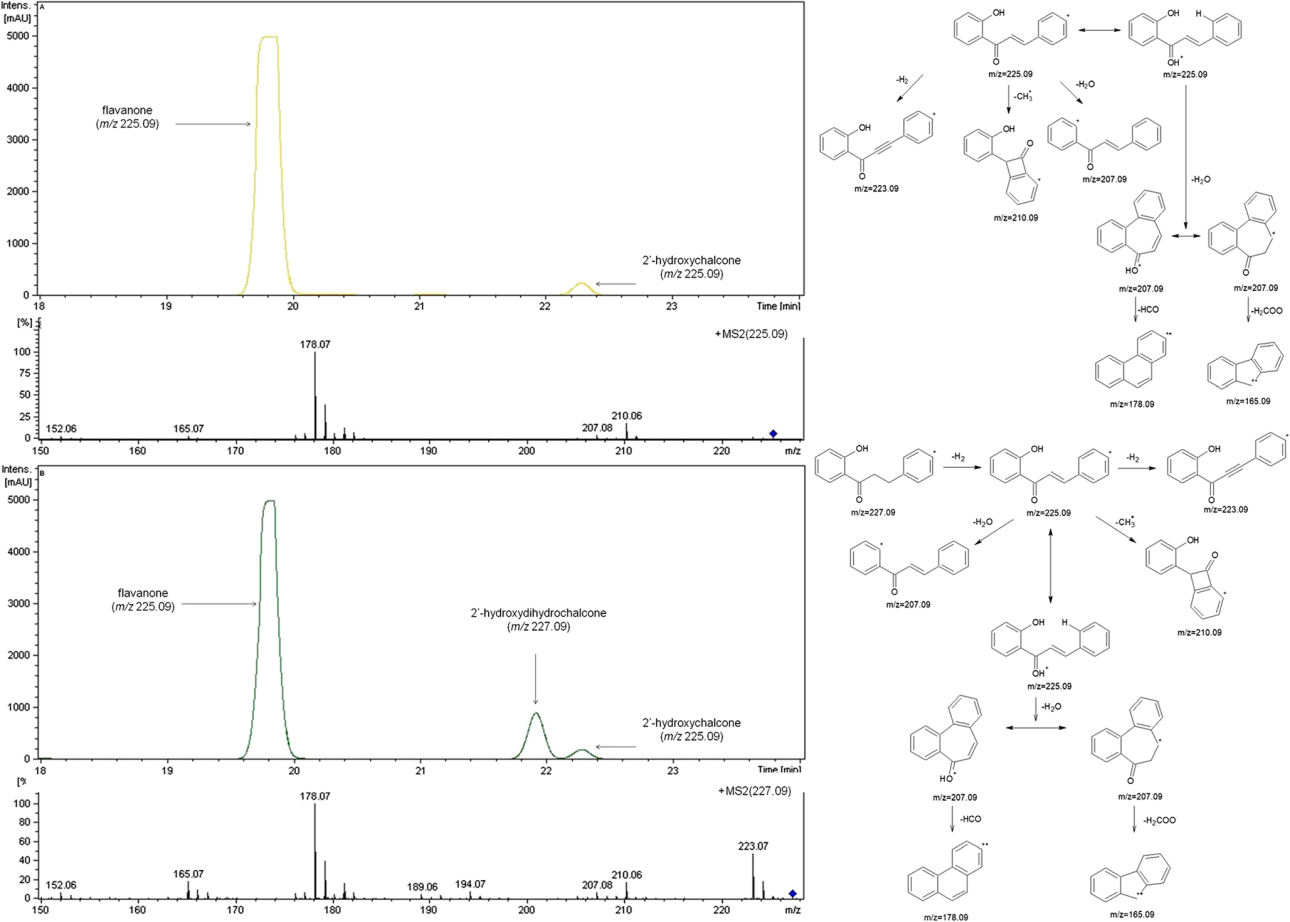
LC-MS profiles of 2′-hydroxychalcone in Z8 medium (substrate control) (**a**) and 2′-hydroxychalcone in culture with *A*. *laxa* (experimental culture) (**b**) obtained after 14 days of incubation. Adequate MS/MS spectra of 2′-hydroxychalcone and flavanone (*m/z* 225.09) and 2′-hydroxydihydrochalcone (*m/z* 227.09) and the proposed general routes of fragmentation of 2′-hydroxychalcone and the corresponding 2′-hydroxydihydrochalcone based on the fragment ions observed in the MS/MS spectra in positive ion mode are also shown

Independent of this chemical rearrangement, all tested (halophilic and freshwater) cyanobacterial species were able to biotransform 2′-hydroxychalcone during the 14 days of incubation to form the same product, albeit with moderate efficiency. The MS/MS spectra of the product, the mass of the molecular ion, its fragmentation pathway and the specific differences compared to the spectrum of 2′-hydroxychalcone clearly suggested the bio-reduction of the propenyl chain and thus the formation of 2′-hydroxydihydrochalcone. The structures of the MS/MS fragment ions in positive ion mode for 2′-hydroxychalcone and 2′-hydroxydihydrochalcone are presented in Fig. 5a, b, along with the proposed pattern of fragmentation.

The retention time, the high agreement with the pattern of specific fragmentation and the compatibility with spectroscopic data published in the literature confirmed that 2′-hydroxydihydrochalcone was obtained as the product of cyanobacterial biotransformation of 2′-hydroxychalcone (Janeczko et al. 2013). This conclusion is supported by the exemplary LC-MS profiles of two samples in Fig. 5a, b: 2′-hydroxychalcone in Z8 medium (substrate control), 2′-hydroxychalcone in the experimental culture of *A*. *laxa* after 14 days of bio-catalytic interactions and the adequate MS/MS spectra of the analysed compounds.

The results of the scaled-up transformation experiments with 2′-hydroxychalcone fully confirmed those obtained at the analytical scale, both with respect to the chemical nature of the products and the dynamics and effectiveness of conversion. Moreover, the experiments performed on the preparative scale yielded sufficient amounts (15–30 mg) of the products for separation by PTLC and for fully credible confirmation of their structures by MS and NMR. The results of NMR experiments (^1^H, ^13^C, HSQC and COSY) performed following preparative (PTLC) separation of the product undoubtedly confirmed the structures of flavanone and 2′-hydroxydihydrochalcone.

The efficiency of the biocatalytic conversions performed by each of the examined strains of cyanobacteria was established based on quantitative determination using liquid chromatography coupled with mass spectrometry LC-MS (Table 1).

**Table 1:** The efficiency of the biocatalytic transformation of the tested hydroxychalcones by cyanobacteria in experiments carried out at preparative scale

Cyanobacterial strain	Substrates			
	2′-hydroxychalcone 1	2″-hydroxychalcone 2	4″-hydroxychalcone 3	
*S*. *platensis*	5 (4.3 ± 0.6)	6 (20.9 ± 2.7)	7 (99.3 ± 0.1)	Product (conversion) [%]
*A*. *laxa*	5 (22.9 ± 0.2)	6 (98.7 ± 0.4)	7 (99.3 ± 0.1)	
*Anabaena* sp.	5 (16.3± 1.0)	6 (97.5 ± 0.3)	7 (31.2 ± 3.1)	
			8 (28.4 ± 1.5)	
			9 (16.2 ± 0.6)	
*A*. *klebahnii*	5 (5.4 ± 0.6)	6 (97.8 ± 0.3)	7 (41.6 ± 4.3)	
			8 (20.0 ± 3.1)	
			9 (22.6 ± 0.7)	
*N*. *moravica*	5 (8.7 ± 0.9)	6 (97.5 ± 1.1)	7 (82.2 ± 0.3)	
			8 (4.4 ± 0.3)	
			9 (6.8 ± 0.0)	
*Ch*. *minutus*	5 (15.6 ± 0.7)	6 (94.0 ± 0.3)	7 (98.5 ± 0.5)	
*M*. *glauca*	5 (6.7 ± 1.0)	6 (75.6 ± 6.8)	7 (46.5 ± 5.6)	
*S*. *aquatilis*	5 (15.8 ± 0.4)	6 (98.6 ± 0.5)	7 (99.1 ± 0.0)	

The moderate yield of biotransformation of 2′-hydroxychalcone into 2′-hydroxydihydrochalcone, which did not exceed 23% for the most effective biocatalyst, *A*. *laxa*, and was less than 5% for *S*. *platensis*, seems to be the result of specific competition between intramolecular rearrangement and the biocatalytic process. The latter was not favourable because, as an enzymatic reaction, it had to be preceded by the transport of the substrate into cyanobacterial cells. Thus, although less effective, the reduction of the double C=C bond of the three-carbon enone linker of the aromatic rings in chalcone molecules tends to be the preferred route of biotransformation of these substances by cyanobacteria.

### Biotransformations of 2″-hydroxychalcone (2)

**Fig. 6:**
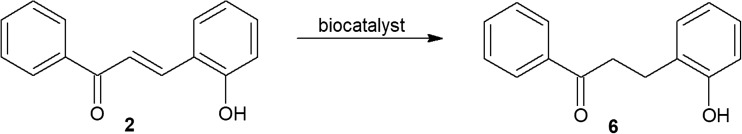
Biotransformation of 2″-hydroxychalcone catalysed by all tested cyanobacterial strains

In contrast to 2′-hydroxychalcone (1), the B-ring substituted 2″-hydroxychalcone (2) was stable in each of the microbial media used. Therefore, its transformation was the result of biocatalytic processes during the 14 days of cyanobacterial culture. Notably, these conversions, independent of the strain, were completely chemoselective, producing only the corresponding 2″-hydroxydihydrochalcone, as shown in Fig. 6. Importantly, no other products were detected by TLC and LC-MS separation.

**Fig. 7:**
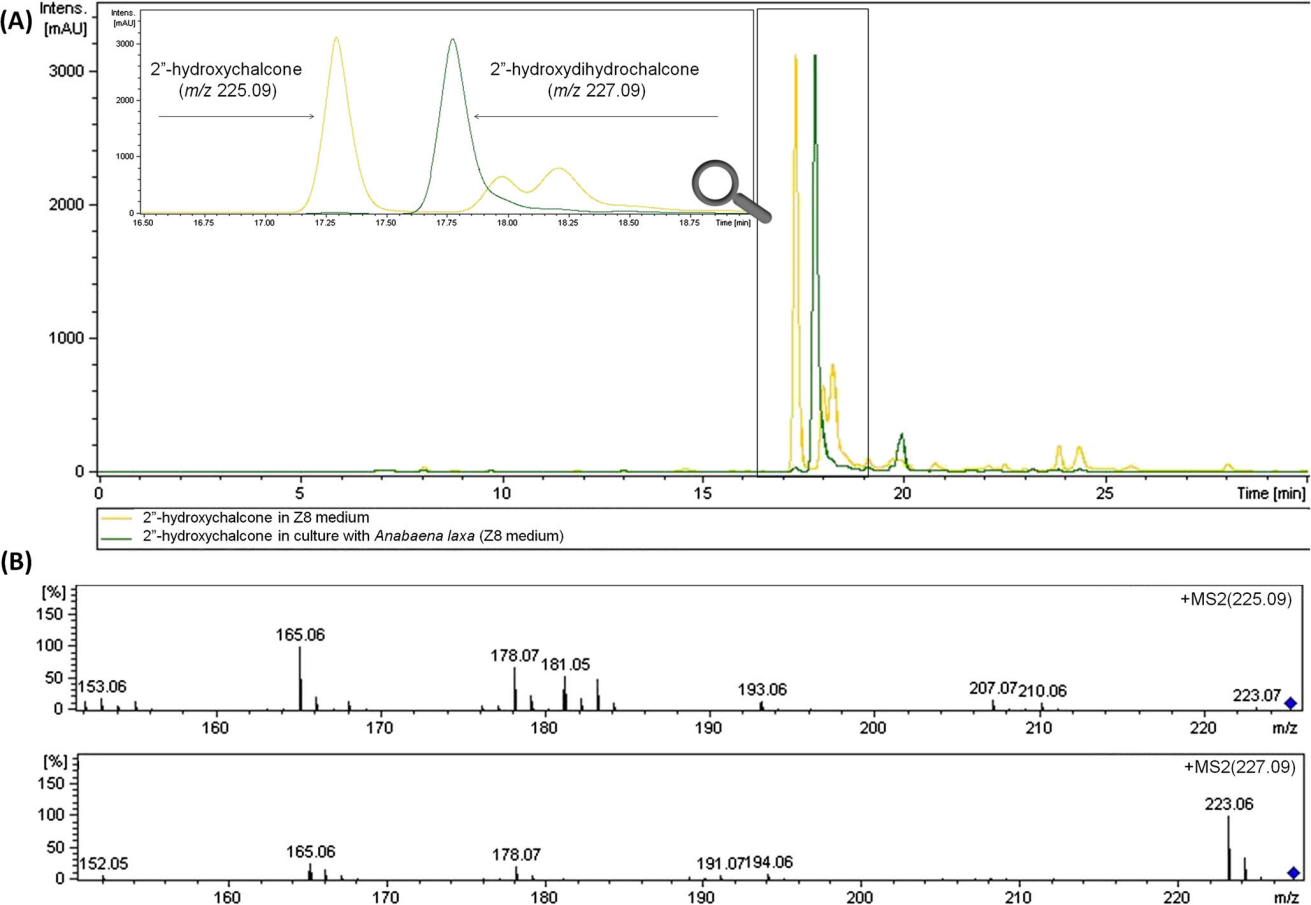
LC-MS profiles prepared after 14 days of incubation of 2″-hydroxychalcone in Z8 medium (substrate control) (**a**) and 2″-hydroxychalcone in the experimental culture of *A*. *laxa* (**b**) and adequate MS/MS spectra of 2″-hydroxychalcone (*m/z* 225.09) and 2″-hydroxydihydrochalcone (*m/z* 227.09)

The LC-MS profiles of the extracted fractions showed that the fraction containing the product after incubation was more homogenous than the corresponding fraction extracted from the substrate control (Fig. 7). We suppose that a type of purification process was responsible for this result; that is, the cyanobacterial cells were able to clean-up the biotransformation medium and somehow absorb some impurities introduced together with the substrate. This ability of the tested cyanobacteria may simplify biotechnological processes associated with these microalgae.

Similar to substrate 1, to confirm the efficiency of conversion and to isolate the product in an amount permitting spectroscopic analysis, a 14-day biotransformation of substrate 2 was conducted on a larger scale. The comparison of the ^1^H NMR, ^13^C NMR and MS spectra of the product with those of the substrate fully confirmed the lack of the double C=C bond of the enone moiety and the presence of the unchanged carbonyl group C=O, thus verifying biocatalytic reduction of this bond as the only observed route of transformation by the examined cyanobacteria.

The bioconversion of 2″-hydroxychalcone proceeded with highly satisfactory efficiency. Most of the tested cyanobacterial strains, including *A*. *laxa*, *Anabaena* sp., *A*. *klebahnii*, *N*. *moravica*, *Ch*. *minutus*, and *S*. *aquatilis*, converted this substrate into its dihydro-derivative in nearly 100% yield (Table 1). Only *M*. *glauca* and *S*. *platensis* formed the product with lower efficiencies of approximately 76 and 21%, respectively.

### Bioconversions of 4″-hydroxychalcone (3)

**Fig. 8:**
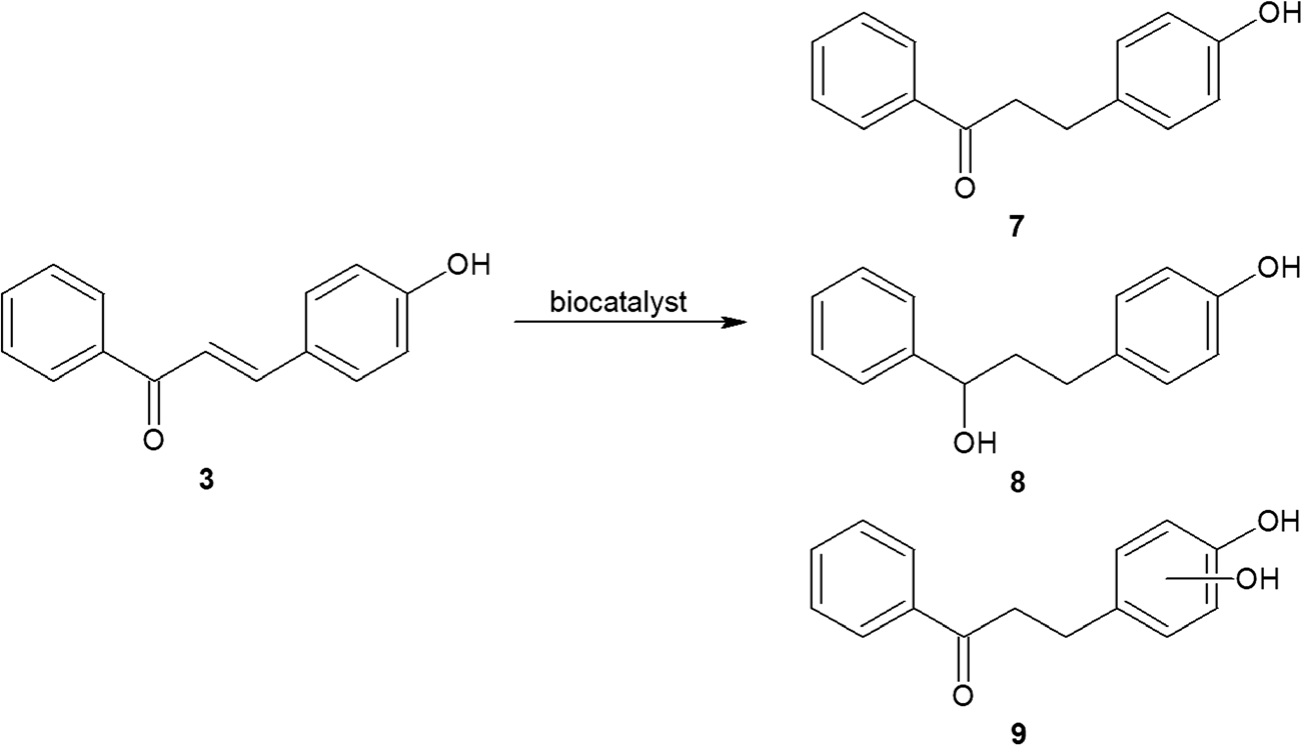
Cyanobacterial transformations of 4″-hydroxychalcone (3) led to the formation of 4″-hydroxydihydrochalcone (7), 4″-hydroxy-1,3-diphenylpropan-1-ol (8) and 4″,x-dihydroxydihydrochalcone (9)

Cyanobacterial transformations of 4″-hydroxychalcone (3) resulted in the increased abundance of three metabolites: 4″-hydroxydihydrochalcone (7), 4″-hydroxy-1,3-diphenylpropan-1-ol (8) and 4″,x-dihydroxydihydrochalcone (9) (Fig. 8).

Only when 4″-hydroxychalcone was used as the substrate were the processes of bioconversion strain dependent. *S*. *platensis*, *A*. *laxa*, *Ch*. *minutus*, *M*. *glauca* and *S*. *aquatilis* hydrogenated only the C=C bond in the three-carbon enone subunit and formed the corresponding dihydrochalcone (Fig. 8). By contrast, the strains *Anabaena* sp., *A*. *klebahnii* and *N*. *moravica* also produced 4″-hydroxy-1,3-diphenylpropan-1-ol (8), and 4″,x″-dihydroxydihydrochalcone (9) (Fig. 8). Notably, the latter three strains belong to the same taxonomic order, Nostocales, whereas the other examined cyanobacteria represent different orders of Cyanophyta.

The LC-MS profiles of the fractions extracted from the cultures of the strains that converted the substrate only to its dihydro-derivative supported this mode of conversion as well as the ability of the cyanobacteria to purify the transformation media to enhance the concentration of this product. Similar to the results for the cultures supplemented with 2″-hydroxychalcone, the activity of these cyanobacteria supports the use of these microorganisms as biocatalysts.

**Fig. 9:**
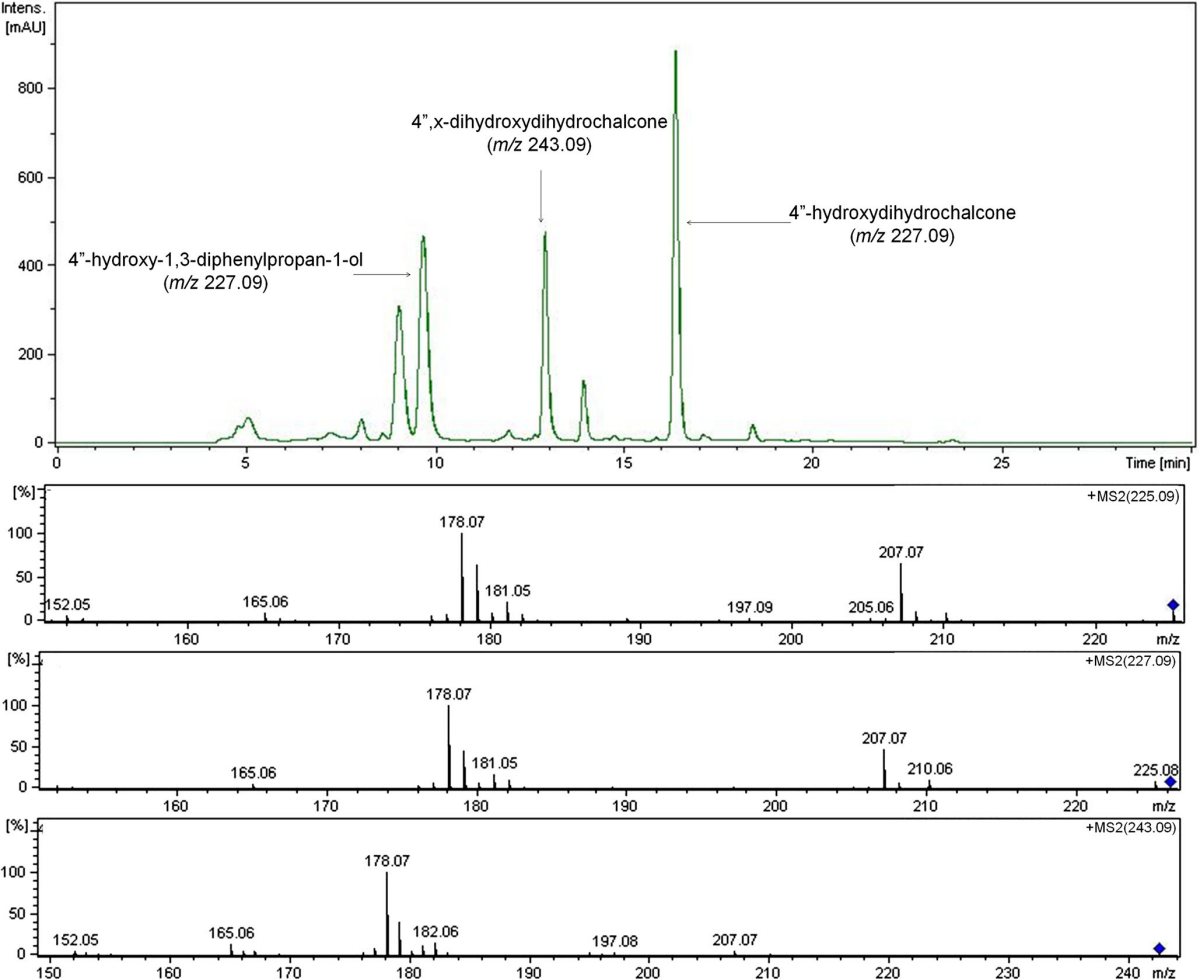
LC-MS profile of the fraction of interest extracted from the culture of *A*. *klebahnii* with 4″-hydroxychalcone after 14 days of incubation and adequate MS/MS spectra of 4″-hydroxychalcone (*m/z* 225.09), 4″-hydroxydihydrochalcone (*m/z* 227.09) and 4″,x-dihydroxydihydrochalcone (*m/z* 243.09)

In contrast to the results above, the LC-MS profiles of the fractions extracted from experimental cultures of the representatives of Nostocales that transformed the substrate into three more abundant products revealed that these strains transformed 4″-hydroxychalcone differently. Nevertheless, even in these cases, the identified products were hydrogenated or additionally hydroxylated derivatives of the substrate. The LC-MS profile of the culture of *A*. *klebahnii* (Fig. 9) is an example that fully reflects this mode of biotransformation. The results for the bioconversion of 4″-hydroxychalcone again confirm our finding of the specific inclination of cyanobacteria to bio-hydrogenate hydroxychalcones.

The spectroscopic data obtained from the analytical cultures were fully confirmed after isolation of the products of bioconversion from the scaled-up (preparative) cultures. The structures of all products were determined by MS, ^1^H NMR and ^13^C NMR. Moreover, the position of the additional hydroxyl group identified as the second hydroxyl substituent in the A/B ring of 4″,x″-dihydroxydihydrochalcone was established more precisely. The HSQC and COSY experiments strongly suggest that this substituent is located at the 3″ or 5″ position, i.e. ‘ortho’ to the initial 4″ hydroxyl group.

Regarding the efficiency of the bioconversion of 4″-hydroxychalcone as a strain-dependent process, it should be emphasised that four (*S*. *platensis*, *A*. *laxa*, *Ch*. *minutus* and *S*. *aquatilis*) of the five cyanobacterial strains transformed the substrate in the most typical way (in this study)—by regiospecific hydrogenation of the enone subunit to form 4″-hydroxydihydrochalcone with > 99% yield (Table 1). By contrast, *M*. *glauca*, which was a less effective biocatalyst for both 2′- and 2″-hydroxychalcones, transformed 4″- hydroxychalcone with 47% yield. This study is the first to report that the halophilic cyanobacterium *S*. *platensis* is a highly effective biocatalyst. In addition, the activities of the cyanobacterial enzymatic systems of *Anabaena* sp., *A*. *klebahnii* and *N*. *moravica* transformed 4″-hydroxychalcone, leading to the formation of three products that were identical for all three strains. These substances were sufficiently abundant to be identified. Importantly, 4″-hydoxydihydrochalcone remained the dominant product, with yields of 31, 42 and 82%, respectively. The less-abundant product, 4″-hydroxy-1,3-diphenylpropan-1-ol, had yields of 28, 20 and 4%, respectively, whereas dihydroxydihydrochalcone was produced in 16, 23 and 7% yield, respectively.

### Spectral data of isolated metabolites

#### Flavanone (4)

^1^H NMR (400 MHz) (DMSO-d^6^) δ (ppm)—7.81 (dd, 1H, H-5), 7.39–7.62 (m, 6H, H-7, H-2′, H-3′, H-4′, H-5′, H-6′), 7.10 (m, 2H, H-6, H-8), 5.68 (dd, 1H, H-2), 3.26 (dd, 1H, H-3), 2.84 (dd, 1H, H-3); ^13^C NMR (100 MHz) (DMSO-d^6^) δ (ppm)—191.63 (C-4), 161.11 (C-8a), 138.95 (C-1), 136.34 (C-7), 128.59 (C-3′, C-5′), 128.57 (C-4′), 126.67 (C-5), 126.36 (C-2′, C-6′), 121.51 (C-6), 120.68 (C-4a), 118.09 (C-8), 78.85 (C-2), 43.54 (C-3); ESI-MS *m/z* 225.0865 [M + H]^+^ (calcd. for C_15_H_12_O_2_ + H, 225.0910); the retention time 19.84 min.

#### 2′-hydroxydihydrochalcone/2′-hydroxy-1,3-diphenylpropan-1-one (5)

As an example, 14-day transformation of 2′-hydroxychalcone **(1)** (80 mg) in the *A*. *laxa* culture yielded 17 mg of compound **5**; ^1^H NMR (400 MHz) (DMSO-d^6^) δ (ppm)—11.87 (s, 1H, OH), 7.95 (dd, 1H, H-6′), 7.51 (ddd, 1H, H-4′), 7.29 (t, 2H, H-3″, H-5″), 7.28 (t, 2H, H-2″, H-6″), 7.18 (t, 1H, H-4″), 6.96 (dd, 1H, H-3′), 6.93 (td, 1H, H-5′), 3.36 (t, 2H, H-3), 2.95 (t, 2H, H-2); ^13^C NMR (100 MHz) (DMSO-d^6^) δ (ppm)—205.21 (C-1), 160.61 (C-2′), 141.03 (C-1″), 136.04 (C-4′), 130.79 (C-6′), 128.44 (C-3″, C-5″), 128.34 (C-2″, C-6″), 125.98 (C-4″), 120.38 (C-1′), 119.26 (C-5′), 117.65 (C-3′), 40.07 (C-3), 29.41 (C-2); ESI-MS *m/z* 227.0948 [M + H]^+^ (calcd. for C_15_H_14_O_2_ + H, 227.1066); the retention times 1–22.26, 4–21.91 min.

#### 2″-hydroxydihydrochalcone/2″-hydroxy-1,3-diphenylpropan-1-one (6)

As an example, transformation of 2″-hydroxychalcone **(2)** (40 mg) in the *A*. *klebahnii* culture yielded 38 mg of compound **6**; ^1^H NMR (400 MHz) (DMSO-d^6^) δ (ppm)—9.39 (s, 1H, OH), 7.97 (dd, 2H, H-2′, H-6′), 7.63 (ddd, 1H, H-4′), 7.52 (t, 2H, H-3′, H-5′), 7.12 (dd, 1H, H-6″), 7.01 (ddd, 1H, H-4″), 6.78 (dd, 1H, H-3″), 6.70 (td, 1H, H-5″), 3.27 (t, 2H, H-3), 2.86 (t, 2H, H-2); ^13^C NMR (100 MHz) (DMSO-d^6^) δ (ppm)—199.64 (C-1), 155.21 (C-2″), 136.61 (C-1″), 133.13 (C-4′), 129.89 (C-6″), 128.75 (C-3′, C-5′), 127.93 (C-2′, C-6′), 127.14 (C-1′), 127.08 (C-4″), 118.91 (C-5″), 114.85 (C-3″), 38.10 (C-3), 24.84 (C-2); ESI-MS *m/z* 227.0948 [M + H]^+^ (calcd. For C_15_H_14_O_2_ + H, 227.1066); the retention times 2–17.31, 5–17.80 min.

#### 4″-hydroxydihydrochalcone/4″-hydroxy-1,3-diphenylpropan-1-one (7)

As an example, transformation of 4″-hydroxychalcone **(3)** (40 mg) in the *Ch*. *minutus* culture yielded 37 mg of compound **7**; ^1^H NMR (400 MHz) (DMSO-d^6^) δ (ppm)—9.14 (s, 1H, OH), 7.95 (dd, 2H, H-2′, H-6′), 7.62 (ddd, 1H, H-4′), 7.51 (t, 2H, H-3′, H-5′), 7.05 (dd, 2H, H-2″, H-6″), 6.65 (dd, 2H, H-3″, H-5″), 3.30 (t, 2H, H-2), 2.82 (t, 2H, H-3); ^13^C NMR (100 MHz) (DMSO-d^6^) δ (ppm)—199.46 (C-1), 155.46 (C-4″), 136.50 (C-1″), 133.32 (C-4′), 131.15 (C-1′), 129.28 (C-3′, C-5′), 128.76 (C-2″, C-6″), 127.96 (C-2′, C-6′), 115.19 (C-3″, C-5″), 39.50 (C-3), 28.76 (C-2); ESI-MS *m/z* 227.0948 [M + H]^+^ (calcd. for C_15_H_14_O_2_+ H, 227.1066); the retention times 3–16.22, 6–16.35 min.

#### 4″-hydroxy-1,3-diphenylpropan-1-ol (8)

As an example, transformation of 4″-hydroxychalcone **(3)** (80 mg) in the *Anabaena* sp. culture yielded 21 mg of compound **8**; ^1^H NMR (400 MHz) (DMSO-d^6^) δ (ppm)—9.14 (s, 1H, OH), 7.30 (d, 4H, H-2″, H-6″, H-2′, H-6′), 7.21 (dt, 1H, H-4′), 6.94 (dd, 2H, C-3′, C-5′), 6.64 (dd, 2H, C-3″, C-5″), 4.48 (dt, 1H, CH-OH), 2.44 (m, 2H, H-2), 1.81 (m, 2H, H-3), 1.23 (s, 1H, OH); ^13^C NMR (100 MHz) (DMSO-d^6^) δ (ppm)—155.21 (C-4″), 146.40 (C-1″), 132.21 (C-1′), 129.06 (C-3′, C-5′), 128.00 (C-2″, C-6″), 126.64 (C-4′), 125.81 (C-2′, C-6′), 115.05 (C-3″, C-5″), 71.98 (C-1), 41.47 (C-3), 30.69 (C-2); ESI-MS *m/z* 229.1103 [M + H]^+^ (calcd. for C_15_H_16_O_2_+ H, 229.1223); the retention times 7–9.68 min.

#### 4″,x″-dihydroxydihydrochalcone/4″,x″-dihydroxy-1,3-diphenylpropan-1-one (9) (x = 3″ or 5″)

As an example, transformation of 4″-hydroxychalcone **(3)** (80 mg) in the *Anabaena* sp. culture yielded 14 mg of compound **9**; ^1^H NMR (400 MHz) (DMSO-d^6^) δ (ppm)—9.23 (d, 1H, OH (3″ or 5″)), 9.08 (d, 1H, OH (4″)), 7.96 (dd, 2H, H-2′, H-6′), 7.62 (ddd, 1H, H-4′), 7.52 (t, 2H, H-3′, H-5′), 7.30 (t, 1H, H-2″ or H-6″), 7.08 (ddd, 1H, H-2″ or H-6″), 6.68 (dd, 1H, H-3″ or H-5″), 3.27 (t, 2H, H-2), 2.86 (t, 2H, H-3); ^13^C NMR (100 MHz) (DMSO-d^6^) δ (ppm)—200.72 (C-1), 158.38 (C-3″ or C-5″), 155.65 (C-4″), 136.52 (C-1″), 133.35 (C-4′), 131.18 (C-1′), 129.25 (C-3′, C-5′), 128.76 (C-2″or C-6″), 128.58 (C-2″or C-6″), 127.93 (C-2′, C-6′), 115.34 (C-3″or C-5″), 39.548 (C-3), 28.72 (C-2); ESI-MS *m/z* 243.0903 [M + H]^+^ (calcd. for C_15_H_14_O_3_ + H, 243.1015); the retention times 8–12.89 min.

## Discussion

The production of a wide range of secondary metabolites is a common adaptation of cyanobacteria to compete successfully in different ecosystems. Because of their phototrophic lifestyle and constant exposure to high oxygen and radical stresses, these biota have a high capability for producing plentiful efficient protective chemicals against oxidative and radical stressors and exhibit adaptive responses to oxidative stresses by stimulating their intrinsic antioxidant defence systems based on mediator compounds, including polyphenolic substances (Babić et al. 2016). Previously published data indicate that the phenolic acids and flavonoids in cyanobacterial cells may be responsible for their antioxidant properties (Singh et al. 2017a), and thus a higher content of phenolics in cyanobacterial species may be presumed as an adaptation strategy of these organisms against abiotic stresses in their specific habitats. Therefore, the properties of these organisms may also include functional values such as free-radical quenching, metal chelation and ROS-scavenging activity (Singh et al. 2017a). Our evaluations are consistent with literature reports showing that metabolites such as flavonoids are involved in the defence system of cyanobacteria against adverse conditions (Singh et al. 2017b).

Independent of the intrinsic content of polyphenolics, the presence of these chemicals outside cyanobacterial cells may significantly influence their growth and metabolism, as demonstrated for naringenin (Żyszka et al. 2017b). This inhibition is higher when the substance is structurally related to hydroxylated chalcones, which are biosynthetic precursors of all flavonoids. We found that the tested hydroxychalcones inhibited the growth of all eight examined strains representing four of five cyanobacterial taxonomic orders, and this inhibition depended more on the structure of the hydroxychalcone than on the microbial strain. Our results are consistent with those obtained by Nakai and co-authors, who observed that the growth of the cyanobacterium *Microcystis aeruginosa* was inhibited in the presence of polyphenols such as ellagic and gallic acids and catechin (Nakai et al. 2005). The presence of flavonoids, such as 4′,5-dihydroxyflavone, apigenin, and luteolin, also suppressed the growth of *M*. *aeruginosa* significantly, and this effect increased with the concentration of these compounds (Huang et al. 2015). Importantly, in the case of our study, the position of the substitution of the hydroxyl group is likely responsible for this effect, suggesting that this aspect is crucial for understanding the mechanism of interactions between chalcones and cyanobacterial species.

Studying mentioned interactions, we observed that each of the tested hydroxychalcones was bioconverted, mainly into the corresponding hydrogenated derivative. Moreover, the processes of (bio)hydrogenation of the C=C bond in the three-carbon enone subunit of the hydroxylated chalcones were regiospecific and mainly proceeded by cyanobacterial transformation. This finding fully corresponds with the results of our previous study on the bio-reduction of chalcones (Żyszka et al. 2017a) and has important ecological implications because hydroxydihydrochalcones have approximately 15–20% lower inhibitory effects on the growth of cyanobacteria (data not presented). Therefore, this route of transformation may be considered a natural component of the cyanobacterial defence system since these organisms successfully faced dozens of extreme environmental stresses during evolution (Żyszka et al. 2017b). This hypothesis is well-supported by the results of the present study because the most active growth inhibitor, 2″-hydroxychalcone, was converted with the highest yields. Thus, we confirmed that the inhibition of the growth of cyanobacterial cells supplemented with hydroxylated chalcones does not affect their activity as biocatalysts. This issue seems to be especially important, since whenever the substrates were available for cyanobacterial catalysis, bioconversion was highly efficient, with yields often above 99%. This finding is notable compared to the chemical hydrogenation of chalcones, which requires 24–48 h in an atmosphere of H_2_ with the use of 10% Pd-C as a catalyst and EtOAc as a solvent. Although dihydrochalcones are obtained in good yields under these conditions (Vijaya Bhaskar Reddy et al. 2017), the efficiency of these processes is far lower than hereby reported 99%, highlighting the value of the presented cyanobacterial catalytic systems. This biotechnological approach does not require chemical catalysts, which may be highly specific and therefore expensive (Luan et al. 2014). For comparison, biotransformation of 2′-hydroxychalcone by yeast strains and filamentous fungi cultures afford the corresponding dihydrochalcone with 2–98% substrate conversion (Janeczko et al. 2013). And transformation of another hydroxylated chalcone, chalconaringenin, in the culture of *Rhodococcus* sp. led to dihydrochalconaringenin (phloretin) in 84% yield (Stompor et al. 2013).

Remarkably lower efficiency of biotransformation of 2′-hydroxychalcone together with its weaker inhibitory action compared to 2″-hydroxychalcone drew our special attention. Basing on the results of our study, we concluded that the reason was the lack of bioavailability of 2′-hydroxychalcone, which tended to intramolecular isomerisation into flavanone. The effective chemical formation of flavanone from the initial chalcone proceeded under mild and totally environmentally friendly conditions (microbial media at room temperature) and thus may be considered a fully ‘green’ process. This rearrangement, however, was not the only side process with unexpected benefits. All examined strains turned out to have a tendency to purify, probably by utilisable absorption, the biotransformation media of small amounts of impurities that accompanied the substrates. In such circumstances, a simplified extraction of pure product together with the mild conditions of the process significantly supports the use of cyanobacterial biocatalysis in the hydrogenation of hydroxychalcones.

The results presented have important implications when compared to those obtained during biotransformations of chalcone published previously (Żyszka et al. 2017a). The same strains of cyanobacteria transformed unsubstituted chalcone to its dihydroderivatives; however, only *A*. *laxa* and *S*. *aquatilis* performed this transformation with high efficiencies similar to those of the corresponding conversion of 4″-hydroxychalcone. In the case of this compound, the routes and efficiency of biohydrogenation and hydroxylation were dependent on the presence and location of the hydroxyl substituent. This information strengthens our conclusions on the structural dependence of the biotransformations of chalcones and their derivatives by cyanobacteria. In this way, these results support the usefulness of these substances as molecular probes for more detailed studies of the enzymes responsible for these transformations. The existence of such enzymes in cyanobacterial cells and their catalytic mechanism has only been postulated (Fu et al. 2013).
